# The relationship between level of autistic traits and local bias in the context of the McGurk effect

**DOI:** 10.3389/fpsyg.2015.00891

**Published:** 2015-06-30

**Authors:** Yuta Ujiie, Tomohisa Asai, Akio Wakabayashi

**Affiliations:** ^1^Information Processing and Computer Sciences, Graduate School of Advanced Integration Science, Chiba UniversityChiba, Japan; ^2^Japan Society for the Promotion of ScienceTokyo, Japan; ^3^NTT Communication Science Laboratories, NTT CorporationKanagawa, Japan; ^4^Faculty of Letters, Chiba UniversityChiba, Japan

**Keywords:** autism spectrum disorder, Autism Spectrum Quotient, the McGurk effect, local bias, individual differences

## Abstract

The McGurk effect is a well-known illustration that demonstrates the influence of visual information on hearing in the context of speech perception. Some studies have reported that individuals with autism spectrum disorder (ASD) display abnormal processing of audio-visual speech integration, while other studies showed contradictory results. Based on the dimensional model of ASD, we administered two analog studies to examine the link between level of autistic traits, as assessed by the Autism Spectrum Quotient (AQ), and the McGurk effect among a sample of university students. In the first experiment, we found that autistic traits correlated negatively with fused (McGurk) responses. Then, we manipulated presentation types of visual stimuli to examine whether the local bias toward visual speech cues modulated individual differences in the McGurk effect. The presentation included four types of visual images, comprising no image, mouth only, mouth and eyes, and full face. The results revealed that global facial information facilitates the influence of visual speech cues on McGurk stimuli. Moreover, individual differences between groups with low and high levels of autistic traits appeared when the full-face visual speech cue with an incongruent voice condition was presented. These results suggest that individual differences in the McGurk effect might be due to a weak ability to process global facial information in individuals with high levels of autistic traits.

## Introduction

Autism spectrum disorder (ASD) has been largely defined in terms of difficulties in social interaction and communication, patterns of repetitive behavior, and narrow interests (American Psychiatric Association, 1994, 2013). In earlier ASD research, the dysfunction of processing information relevant to social interaction was the main focus of investigations. It has been revealed that individuals with ASD show different patterns in face perception (e.g., Deruelle et al., 2004) and emotion recognition (e.g., Baron-Cohen et al., 2001a) compared to individuals with typical development (TD). In addition to dysfunction in processing visual stimuli, recent studies have shown that individuals with ASD exhibit atypical processing in audio-visual speech perception (Massaro and Bosseler, 2006; Smith and Bennetto, 2007), which indicates a limited ability to integrate visual and auditory information. This dysfunction is considered to lead to communication impairment in ASD because speech perception is one of the core functions of face-to-face communication.

In face-to-face communication with others, we realize what another person is saying through the processing of audio-visual speech information. An early study of audio-visual speech perception provided strong evidence that visual information improves the auditory speech percept (Sumby and Pollack, 1954). A classic example that demonstrates the interaction between hearing and vision in speech perception is the McGurk effect (McGurk and MacDonald, 1976). This effect may be experienced when the visual shape produced during speech of a phoneme (e.g., /ga/) is dubbed with a sound recording of a different phoneme (e.g., /ba/), which often causes a third, intermediate phoneme (e.g., /da/) to be perceived. Similarly, for the monosyllabic combination of the visual /ka/ and auditory /pa/, participants often reported hearing /ta/.

Abnormal processing of audio-visual speech integration in individuals with ASD has been reported in a number of studies (De Gelder et al., 1991; Williams et al., 2004; Iarocci et al., 2010; Taylor et al., 2010; Saalasti et al., 2011, 2012; Woynaroski et al., 2013; Stevenson et al., 2014). Iarocci et al. (2010) reported that children with ASD showed less visual influence and more auditory influence during bimodal speech perception than controls did, due to poor lip-reading ability, and this finding was supported by Williams et al. (2004). On the other hand, De Gelder et al. (1991) reported that children with ASD are less influenced by the auditory percepts from visual speech cues, although they did not differ in lip-reading ability from children with TD. Similar results have been reported in children with ASD (Stevenson et al., 2014) and in adults with ASD (Saalasti et al., 2011, 2012). Although, the results are mixed, it has often been reported that individuals with ASD exhibit a weak degree of visual influence on perceiving a voice during audio-visual speech perception.

Another study suggested that the reduced McGurk effect in individuals with ASD meant that there was a delay, rather than a deficit, in the development of audio-visual integration (Taylor et al., 2010). Taylor et al. (2010) showed that younger children with ASD exhibit delayed visual accuracy and audio-visual integration (the McGurk effect) compared to children with TD, but appeared to catch up with their TD peers in the older age ranges. In line with this, Keane et al. (2010) revealed no individual differences in the McGurk effect between adults with and without ASD. This was inconsistent with the results in some previous studies (Saalasti et al., 2012; Stevenson et al., 2014).

Neuropsychological data provide us with some advantages to identify whether individuals with ASD show weaker visual influence on perceiving a voice. In ASD, several studies have reported anatomical and functional abnormalities in the superior temporal sulcus (STS) (see, for a review, Zilbovicius et al., 2006; Redcay, 2008). The STS is critical for integration of auditory and visual speech information, and influences the likelihood of the McGurk effect occurring (Calvert et al., 2000; Nath and Beauchamp, 2011). Redcay (2008) argued that impairments in STS function might lead to abnormalities in speech perception in individuals with ASD. In the sample of individuals with TD, a functional magnetic resonance imaging (fMRI) study revealed a significant positive correlation between the likelihood of perceiving the McGurk effect and the amplitude of the response in the left STS (Nath and Beauchamp, 2012). This means that individuals with a weak response in the left STS showed fewer instances of the McGurk effect when they observed audio-visual-incongruent stimuli. Another study using near-infrared spectroscopy (NIRS) reported a significantly negative correlation between level of autistic traits and regional cerebral blood volume in the left STS during face-to-face conversation among adults with TD (Suda et al., 2011). These studies led us to hypothesize that individuals with ASD (or a high level of autistic traits) might show fewer instances of the McGurk effect due to a weak response in the left STS.

The reason why previous results are mixed might be due to the heterogeneity of the clinical population. For instance, abnormalities in sensory inputs (hyper- or hypo-sensitivity), which is one of the core symptoms of ASD, have been found in more than 90% of individuals with ASD in at least one sensory domain (Tomchek and Dunn, 2007; Crane et al., 2009), but in which sensory domain the abnormality appears varies (e.g., visual: Simmons et al., 2009; auditory: Haesen et al., 2011; O'Connor, 2012; tactile: Foss-Feig et al., 2012). With regard to the McGurk effect, one study showed that a fusion response was correlated with the degree of auditory processing difficulty, as assessed by the Sensory Profile (Dunn and Westman, 1997) among individuals with ASD (Woynaroski et al., 2013). Saalasti et al. (2012) examined the distribution of the likelihood of the McGurk response occurring and showed that the difference between the control group and the clinical group with ASD was significant. Mixed results in previous studies might be due to the heterogeneity in the profile of hyper- or hypo-sensitivity, which is difficult to control in a clinical group.

An analog design is one approach used to study ASD symptoms among individuals with TD, by examining the relationship between level of autistic traits and performance on a cognitive or perceptual task. This approach is based on the dimensional model of ASD, which assumes that autistic traits are distributed on a continuum over clinical and general populations (Frith, 1991; Baron-Cohen, 1995). In order to assess the degree of autistic traits in any individual adult with a normal intelligence quotient, Baron-Cohen et al. (2001b) developed the Autism Spectrum Quotient (AQ). The AQ is a self-report questionnaire and is useful as a screening scale to not only distinguish between clinical and control groups but also measure the distribution of autistic traits within the general population. The validity and reliability of this screening scale have been confirmed in various countries (UK: Baron-Cohen et al., 2001b; the Netherlands: Hoekstra et al., 2008; Australia: Lau et al., 2013; and Japan: Wakabayashi et al., 2006b). Moreover, our pilot study (Ujiie and Wakabayashi, 2015) found that the overlap between level of autistic traits and the degree of hyper- or hypo-sensitivity, which was assessed by the Glasgow Sensory Questionnaire (Robertson and Simmons, 2012), was small among a general population. Because an analog design allows for control of the heterogeneity in the profile of hyper- or hypo-sensitivity, we adopted this design to examine the relationship between level of autistic traits and McGurk effects among a population with TD who were free from problems with sensory inputs.

The purpose of this study was to use an analog design based on the dimensional model of ASD to investigate the relationship between individual differences in the McGurk effect and autistic traits in the general population. First, we investigated whether autistic traits were correlated with a weaker visual influence on speech perception under a without-noise condition (Experiment 1). As the McGurk stimuli, we used the combination of auditory /pa/ and visual /ka/ stimuli, because this combination was likely to be perceived as a stronger illusion than other combinations (e.g., auditory /ba/ and visual /ga/ stimuli) in a Japanese sample (Sekiyama, 1994). There were three possible responses to the McGurk stimuli, which were as follows: audio response (/pa/ response), fused response (/ta/ response), and visual response (/ka/ response). In this study, we defined the rate of fused response, which was the frequency of the McGurk effect occurring, as the degree of visually captured percept when hearing and viewing the McGurk stimuli. We defined the rate of /pa/ response, which was the correct response to the audio-visual-incongruent stimuli, as the strength of visual influence on perceiving a voice. Thus, we hypothesized that the degree of autistic traits would correlate negatively with the rate of fused response and correlate positively with the rate of audio response in the context of the McGurk stimuli. In Experiment 2, we focused on the local bias toward visual speech cues in individuals with ASD, and investigated whether this bias underlies the link between level of autistic traits and the McGurk effect.

Individuals with ASD have been shown to tend to prefer local over global information when presented multiple information sources (Happe and Frith, 2006). Such cognitive specificity in individuals with ASD is called the local bias (Frith, 1991; Happe and Frith, 2006), and this bias has been mainly reported in relation to visuospatial tasks. For instance, individuals with ASD show better performance than individuals with TD on the Embedded Figures Test (EFT; Brosnan et al., 2012), in which one is required to find a local (embedded) target within a global context constructed of multiple figures. A similar result has been found in the Navon-type Global–Local Naming Task (Reed et al., 2011) and various perceptual and cognitive tasks (Happe and Frith, 2006).

Furthermore, the local bias has been found in face processing tasks (Joseph and Tanaka, 2003; Deruelle et al., 2004; Kätsyri et al., 2008). Deruelle et al. (2004) investigated whether children with ASD preferred to use local (high spatial frequency) rather than global (low spatial frequency) information during a face matching task. The results showed that children with ASD showed better performance when using local information than when they used global information. Kätsyri et al. (2008) found a similar result in the recognition of dynamic facial emotions among adults with ASD. In addition, some studies suggested a preference of gaze toward the mouth region when individuals with ASD perceived face stimuli (Klin et al., 2002; Joseph and Tanaka, 2003). Joseph and Tanaka (2003) revealed that individuals with ASD use more information from around the mouth region of face stimuli in face recognition tasks. Klin et al. (2002) showed that individuals with ASD tend to gaze at the mouth region during the viewing of conversation. These results indicate that individuals with ASD might prefer to use local information, particularly that from the mouth region, during face processing. Individuals with high AQ scores who exhibit a local bias (Reed et al., 2011) might be less likely to experience the McGurk effect. To clarify this, we should examine whether face processing influences the occurrence of the McGurk effect, in general.

Whether face processing is needed for audio-visual speech perception has been discussed in the past. According to Bruce and Young's (1986) model, face perception has three important functions, comprising recognition of facial identity, facial expression, and facial speech. In line with this suggestion, some studies have demonstrated a relationship between face processing and speech perception. De Gelder et al. (1991) showed that face identification correlates positively with the influence of lip-reading on audio-visual-incongruent stimuli. Rosenblum et al. (2002) showed that face processing and speech perception share the same dynamic information. Some studies, however, suggested that a mouth-only presentation influenced voice perception and produced the McGurk effect as well as a whole-face presentation did (Rosenblum et al., 2000; Hietanen et al., 2001). Other studies showed the role of extraoral facial information in audio-visual speech perception (Thomas and Jordan, 2004; Jordan and Thomas, 2011). Jordan and Thomas (2011) revealed that occluded oral areas disrupted performance but that observers could use lip-reading and observe visual speech influences from extraoral areas. Thomas and Jordan (2004) showed that an extraoral movement during visual speech was effective to perceive visual speech cues and influenced audio-visual speech perception.

The role of holistic face processing on speech perception has been investigated by using methods to examine the effect of holistic processing on face perception. For instance, some studies investigated the face inversion effect, which is a phenomenon that causes difficulty in holistic face processing by showing an inverted face, in the context of audio-visual speech perception (Jordan and Bevan, 1997; Rosenblum et al., 2000; Eskelund et al., 2015). One study showed a robust effect (Eskelund et al., 2015) while another study found only a partial effect (Jordan and Bevan, 1997), which means that the face inversion effect depends on the stimulus. Rosenblum et al. (2000) examined the role of holistic facial information in audio-visual speech perception, compared to full-face Thatcher-type speech stimuli and inverted mouth-alone speech stimuli. In their study, the full-face Thatcher-type stimuli were created by combining an upright face with an inverted mouth visual speech cue. They reported that only the full-face Thatcher-type stimuli disrupted voice perception for the audio-visual-incongruent condition (the combination of auditory /va/ and visual /ba/). This result, which is called the McThatcher effect, was replicated in Eskelund et al. (2015). Hietanen et al. (2001) investigated the effect of facial configuration context on the McGurk effect. They manipulated the location of facial features in visual stimuli, using either a natural or scrambled location. In their results, only an asymmetrically scrambled face disrupted the likelihood of the McGurk effect, but this effect depended on the stimulus. They concluded that facial configuration information can be used in audio-visual speech perception, although this information is not necessary. These studies indicate that processing of global (holistic) visual speech cues might influence the occurrence of the McGurk effect.

In summary, based on the dimensional model of ASD, we administered two experiments to examine the relationship between level of autistic traits and McGurk effects in university students. In Experiment 1, we investigated the correlation between level of autistic traits and individual differences in audio-visual speech perception. We hypothesized that individuals with high levels of autistic traits would show a reduced likelihood of the McGurk effect occurring than would individuals with low levels of autistic traits. In Experiment 2, we examined whether the local bias toward visual speech cues modulates individual differences in the McGurk effect, by manipulating presentation types of visual stimuli (parts of the face or the full face). With regard to the likelihood of the McGurk effect occurring, we hypothesized that the visual influence on voice perception would be greater in the full-face presentation condition than in the partial-face presentation condition. In addition, we hypothesized that individual differences in the McGurk effect would appear when the full-face visual speech cue with an incongruent voice condition was presented, because of the local bias toward visual speech cues in individuals with high levels of autistic traits. The outcomes from these experiments will allow us to understand the effect of face processing on speech perception, and how audio-visual speech integration in individuals with ASD functions from an analog perspective.

## Experiment 1

In Experiment 1, we investigated the correlation between AQ scores and level of accuracy for perceiving audio-visual stimuli and auditory stimuli, and assessed the likelihood of the McGurk effect (the rate of /ta/ response) occurring among non-ASD university students. For audio-visual-incongruent stimuli, we hypothesized that, because of the weak visual influence of perceiving a voice, the AQ scores would correlate negatively with the likelihood of the McGurk effect occurring, and positively with the rate of the /pa/ response being reported.

### Methods

#### Participants

Participants were 46 university students (12 males and 34 females) who were recruited from an introductory psychology class at Chiba University. The mean age of the participants was 19.4 years (*SD* = 3.56). All participants were native speakers of Japanese and reported normal hearing and vision. They provided written informed consent in the class, and took part voluntarily in this experiment. After the experiment, they received an oral debriefing.

#### Stimuli

##### Japanese version of the AQ

The AQ was normalized for use in the Japanese population by Wakabayashi et al. (2006b). The AQ contains 50 items for assessing the following five domains: social skill, attention switching, attention to detail, communication, and imagination. Participants rate each item on a 4-point response scale from “agree” to “disagree.” Each item is scored 0 or 1 point according to the scoring manner described in previous studies (Baron-Cohen et al., 2001b; Wakabayashi et al., 2006b), so that total scores on the AQ range from 0 to 50.

##### Audio-visual task

The audio-visual stimuli were created from simultaneous audio and video recordings of six Japanese speakers' utterances (three female). The visual stimuli were speakers' faces recorded using a digital video camera (GZ-EX370, JVC KENWOOD). The audio stimuli were the utterances (/pa/, /ta/, or /ka/) collected using a dynamic microphone (MD42, SENNHEISER). The video clip (720 × 480 pixels, 29.97 frames/s) and the speech sound (digitized at 48,000 Hz, with 16-bit quantization resolution) were combined and synchronized using Adobe Premiere Pro CS6. The mean duration of the audio-visual stimuli was 1.2 s.

There were three stimulus conditions, comprising audio-only (e.g., auditory /pa/), audio-visual congruent (e.g., auditory /pa/, visual /pa/), and audio-visual incongruent (e.g., auditory /pa/, visual /ka/). Each condition included 18 trials per block.

In the audio-only condition, the audio stimuli (/pa/, /ta/, or /ka/) were presented without the visual stimuli. In the audio-visual-congruent condition, all three combinations of the audio and visual stimuli were presented. In the audio-visual-incongruent condition, the combination of the auditory /ka/ stimulus dubbed with visual /pa/ was excluded, because the percept (e.g., /pka/) caused by this combination stimulus is not a Japanese native syllable. Therefore, the voice (/pa/) and video (/ka/) combined stimuli were presented three times per block to make the same number of audio-visual-congruent trials.

#### Apparatus

The experiment was conducted using Hot Soup Processor Version 3.3 (Onion software). The video signals were presented on a 19-inch cathode ray tube (CRT) monitor (E193FPp, Dell), and the speech sound was presented through a headphone (MDR-Z500, Sony) at approximately a 65 dB sound pressure level, adjusted using a mixing console (MW8CX, Yamaha).

#### Procedure

Participants were seated at a distance of approximately 50 cm from the CRT monitor, wearing the headphone. Participants were instructed to report what they heard (/pa/, /ta/, or /ka/) by a key press. In each trial, a fixation point was displayed for 1000 ms at the center of the CRT monitor, followed by either the congruent or the incongruent stimulus. Then, a blank display was presented until participants responded.

The first block included 18 congruent stimuli and 18 incongruent stimuli. The second block included 18 auditory stimuli. All participants completed both blocks after undergoing six practice trials each. The order of trials was randomized for each block. After all of the tasks were finished, participants completed the questionnaire.

#### Data analysis

Statistical analysis was conducted using R version 2.15.2 for Windows (R Foundation for Statistical Computing, Vienna, Austria). To examine the effect of stimuli conditions, we analyzed the mean accuracies for each condition using a One-Way analysis of variance (ANOVA), with conditions as a within-participants factor. The likelihood of the McGurk effect occurring was analyzed in the audio-visual-incongruent condition using a chi-square test. The relationship between task performance and AQ scores was analyzed using Pearson correlation coefficients. In addition, group differences between a high-AQ group and a low-AQ group were analyzed using independent samples *t*-tests for each condition.

### Results

Table 1 shows mean accuracies for the audio-visual-congruent condition and the audio-only condition, and the mean response rate for the audio-visual-incongruent condition. A One-Way ANOVA with conditions as a within-participants factor revealed a main effect of conditions, *F*_(2, 90)_ = 197.215, *p* < 0.01, partial η^2^ = 0.81. Multiple comparisons (Holm method) showed that accuracies for correctly perceiving the voice in the audio-only condition (*M* = 97.3%) and the audio-visual-congruent condition (*M* = 98.1%) were higher than in the audio-visual-incongruent condition (*M* = 34.9%; *p* < 0.05). However, the accuracy in the audio-only condition did not differ from that in the audio-visual-congruent condition. In the audio-visual-incongruent condition, the rate of the /ta/ response (*M* = 61.1%) was higher than the rate of the /pa/ response (*M* = 34.9%; χ^2^ = 7.66, *p* < 0.01) and the /ka/ response (*M* = 4.0%; χ^2^ = 20.33, *p* < 0.01), which confirmed the occurrence of the McGurk effect.

**Table 1:** **Mean and standard deviations (SD) of response rate in all conditions**.

	**Mean**	**SD**	**Correlations with the AQ scores**
**AUDIO-ONLY CONDITION**			
Correct response (/pa/, /ta/, /ka/)	0.97	0.03	0.00
**AUDIO-VISUAL-CONGRUENT CONDITION**			
Correct response (/pa/, /ta/, /ka/)	0.99	0.03	−0.10
**AUDIO-ONLY-INCONGRUENT CONDITION**			
Audio response (/pa/)	0.35	0.29	0.29^*^
Fused response (/ta/)	0.61	0.28	−0.30^*^
Visual response (/ka/)	0.04	0.08	−0.19

Also displayed are correlations between the AQ scores and response rates for experiment 1. N = 46. AQ, Autism-spectrum Quotient. Correct response (/pa/, /ta/, /ka/): Mean correct responses for all stimuli in the audio-visual-congruent condition or the audio-only condition.

* p < 0.05,

** p < 0.01.

The AQ scores ranged from 10 to 37 (*M* = 20.8, *SD* = 5.42). The distribution of AQ scores in this sample was slightly higher than that reported in the original publication of the AQ (Baron-Cohen et al., 2001b). To examine the relationship between task performances and the AQ scores, we calculated Pearson correlation coefficients (Table 1). No significant correlation was observed for the audio-visual-congruent condition or the audio-only condition. For the audio-visual-incongruent condition, the AQ was significantly positively correlated with the /pa/ response, *r*_(46)_ = 0.29, *p* < 0.05, and significantly negatively correlated with the /ta/ response, *r*_(46)_ = −0.32, *p* < 0.05. These correlations suggest that individuals with low AQ scores show a more visually captured response and less audio response than individuals with high AQ scores do.

**Figure 1:**
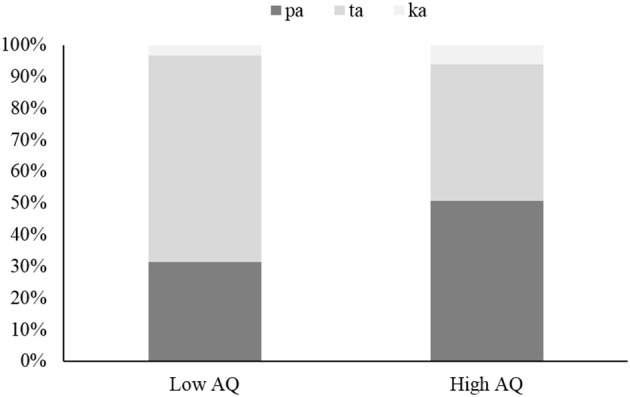
**The response rate for each audio-visual-incongruent stimulus in the low-AQ group and the high-AQ group**. Possible responses to the stimuli were audio response (/pa/ response), fused response (/ta/ response), and visual response (/ka/ response).

Next, we examined group differences between individuals with high AQ scores and those with low AQ scores in each condition. From among the participants, we picked eight with scores of 15 or under (mean AQ – 1 SD), and another eight with scores of 26 or over (mean AQ + 1 SD). We regarded the former as the low-AQ group (4 males and 4 females, mean AQ = 13.5) and the latter as the high-AQ group (3 males and 5 females, mean AQ = 29.3). A between-groups *t*-test showed a significant difference in the AQ scores, *t*_(14)_ = 11.26, *p* < 0.01, *r* = 0.95. Similarly, we conducted independent samples *t*-tests for each condition. No significant difference was found in the audio-only condition, *t*_(14)_ = 1.17, *ns*, *r* = 0.29, or in the audio-visual-congruent condition, *t*_(14)_ = 0.43, *ns*, *r* = 0.12 (see Supplementary Material). For the audio-visual-incongruent condition (see Figure 1), the rate of the /ta/ response was higher in the low-AQ group (*M* = 65.3%) than in the high-AQ group (*M* = 43.1%). This difference was marginally significant, *t*_(14)_ = 1.79, *p* < 0.10, *r* = 0.43; however, the rate of the /pa/ response was not significantly different, *t*_(14)_ = 1.62, *p* = 0.12, *r* = 0.40. These results indicate that individuals with high AQ scores show weaker visually captured responses than individuals with low AQ scores do, although accuracies for perceiving voice and audio-visual speech did not differ.

### Discussion

In this experiment, we investigated the relationship between audio-visual speech integration and the level of autistic traits in healthy students. We found that the level of autistic traits correlated negatively with the rate of fused response and positively with the rate of audio response in the audio-visual-incongruent condition. Moreover, the results revealed that individuals with high AQ scores showed a weaker fused response than individuals with low AQ scores did, although there was no significant difference in the audio response rate. On the other hand, neither significant correlations nor group differences were found in the audio-visual-congruent condition and audio-only condition. These results indicate that individuals with higher levels of autistic traits tended to show a weaker visual influence on perceiving a voice when processing audio-visual-incongruent speech information.

Several studies reported that individuals with ASD showed a weaker visual influence only when McGurk stimuli are presented (e.g., De Gelder et al., 1991). This study replicated those results in a sample of university students. As we hypothesized, our results indicate that the weakness of visual influence on audio-visual speech perception exists along the distribution of AQ in the general population. This finding might support the dimensional model of ASD, because individuals with high AQ scores in this study and individuals with ASD in previous studies (e.g., De Gelder et al., 1991) showed a similar tendency when processing audio-visual-incongruent speech.

However, in this experimental task, it was not clear what factor led to a weaker visual influence on audio-visual speech perception. One possibility is that the local bias toward visual speech cues reflected individual differences in the McGurk effect. There was a local bias effect of cognitive specificity on individuals with ASD, meaning that there is a bias toward processing local information in preference to global information (Frith, 1991; Happe and Frith, 2006). In addition to the results for visuo-spatial tasks (Reed et al., 2011; Brosnan et al., 2012), recent studies have reported that individuals with ASD have a preference for feature-based processing of face stimuli (Joseph and Tanaka, 2003; Deruelle et al., 2004), and for focusing on the local (mouth) region during the viewing of conversation videos (Klin et al., 2002). Some studies have suggested that the influence of visual speech cues in processing audio-visual-incongruent stimuli is related to the processing of faces (De Gelder et al., 1991), especially in the global (holistic) facial context (Rosenblum et al., 2000). Thus, if global facial context enhances the influence of visual speech cues on perceiving a voice, individual differences in the McGurk effect between individuals with high AQ scores and those with low AQ scores might be due to a weak ability to process global facial information in McGurk stimuli. To confirm this, we conducted Experiment 2.

## Experiment 2

In Experiment 2, we manipulated presentation types of visual stimuli to examine whether the local bias affected individual differences in the McGurk effect. We set two stimulus conditions, i.e., the audio-visual-congruent condition and the audio-visual-incongruent condition. For the audio-visual-incongruent condition, we defined the rate of fused responses as the frequency of visually captured percept, while we defined the rate of /pa/ responses as the strength of visual influence to perceiving voice, as in Experiment 1.

In addition, we created the following four types of visual stimuli: no image (audio-only), mouth-only, eyes and mouth, and full face. Only the full-face stimuli included global facial information of visual speech cues. For the audio-visual-incongruent condition, we hypothesized that audio response would be observed less frequently in the full-face presentation than in the other stimuli conditions if the processing of global visual speech cues is related to the degree of visual influence on perceiving a voice. Moreover, we also hypothesized that the differences between individuals with high AQ scores and those with low AQ scores would diminish (or become small) when a voice and an incongruent visual speech cue without global visual information, such as only the mouth region, was presented.

### Methods

#### Participants

Another 50 healthy students (12 males and 38 females), who were recruited from an introductory psychology class at Chiba University, participated in the experiment. The mean age of the participants was 19.4 years (*SD* = 3.41). All participants were native speakers of Japanese and reported normal hearing and normal (or corrected) vision. They provided written informed consent in the class and took part in the study voluntarily. After the experiment, they received an oral debriefing.

#### Stimuli

We used the same stimuli as in Experiment 1, comprising six (3 females) Japanese speakers' utterances of three syllables (/pa/, /ta/, or /ka/). There were two audio-visual stimulus conditions, i.e., the audio-visual congruent and audio-visual incongruent. The audio-visual stimuli consisted of a congruent auditory /pa/–visual /pa/, a congruent auditory /ta/–visual /ta/, a congruent auditory /ka/–visual /ka/, and an incongruent auditory /pa/–visual /ka/.

**Figure 2:**
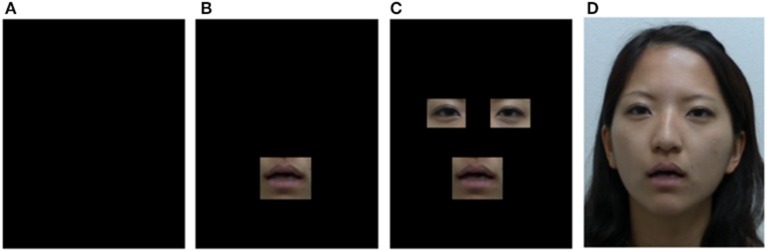
**Examples of the four types of visual stimuli used in Experiment 2**. **(A)** No image (audio only). **(B)** Mouth-only presentation. **(C)** Eyes and mouth presentation. **(D)** Full-face image presentation. All of these images were presented with a congruent or incongruent voice in the experiment.

The four types of presentations of visual stimuli—no image (audio-only), mouth-only, eyes and mouth, and full face—(examples of the visual stimuli are shown in Figure 2) were created for each condition by using Adobe Premiere Pro CS6 to crop eye regions and the mouth region from visual images. The eye region included the region from the inner corner of the eyes to the outer corner. The mouth region included a range of motion of the upper lip and lower lip. This task consisted of 72 congruent stimuli and 24 incongruent stimuli per block.

Following the experimental tasks, we used the Japanese version of the AQ (Baron-Cohen et al., 2001b; Wakabayashi et al., 2006b) to measure the level of autistic traits in the participants.

#### Procedure

The experiment was carried out individually, using the same apparatus as in Experiment 1. Participants were seated at a distance of approximately 50 cm from the 19-in CRT monitor, wearing the headphone. They were instructed to report what they heard (/pa/, /ta/, or /ka/) by pressing buttons on a keyboard. In each trial, a fixation point was displayed for 1000 ms at the center of the CRT monitor. After that, either the congruent or the incongruent stimulus was presented, followed by a blank display presented until participants responded. All participants completed the two blocks of the main session after undergoing the 10-trial practice session. The order of trials was randomized for each block. After the tasks were finished, participants completed the questionnaire.

#### Data analysis

Statistical analysis was conducted using R version 2.15.2 for Windows (R Foundation for Statistical Computing, Vienna, Austria). In order to examine the effect of visual presentation type and stimulus condition, rates of correct (audio) responses were analyzed using a Two-Way ANOVA with visual presentation types and stimulus conditions as within-participant factors. As in Experiment 1, the relationship between the AQ scores and task performance was analyzed using Pearson correlation coefficients for each stimulus condition. In addition, group differences between high- and low-AQ groups were analyzed using a mixed ANOVA with visual presentation as a within-participant factor and groups as a between-participants factor for rates of correct responses in the audio-visual-congruent condition and of audio responses in the audio-visual-incongruent condition.

### Results

**Figure 3:**
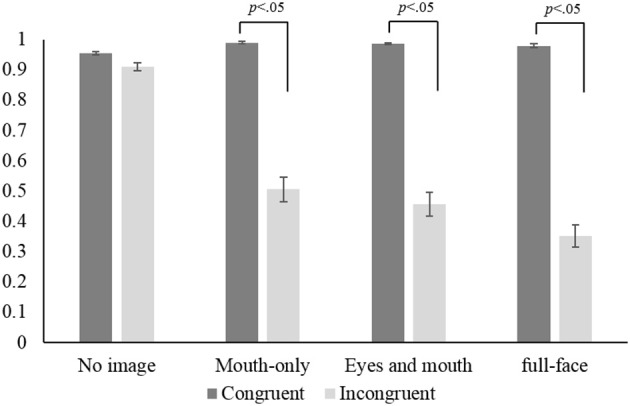
**Mean accuracy for each condition across all participants**. In the audio-visual-congruent condition, mean accuracy is the mean rate of correct responses for the three syllables. In the audio-visual-incongruent condition, mean accuracy is the rate of audio response. Error bars indicate standard errors.

Figure 3 summarizes mean accuracies for the congruent and incongruent stimuli conditions in all types of visual presentation. A Two-Way ANOVA with visual presentation types and stimulus conditions as within-participant factors revealed main effects of stimulus conditions, *F*_(1, 49)_ = 201.10, *p* < 0.01, partial η^2^ = 0.80, and visual presentation, *F*_(3, 149)_ = 110.72, *p* < 0.01, partial η^2^ = 0.69, and a significant one-way interaction, *F*_(1, 49)_ = 132.10, *p* < 0.01, partial η^2^ = 0.73. Multiple comparisons (Holm method) showed that the accuracy for no image, which presented only the auditory stimulus, was lower than for the other types of presentation in the audio-visual-congruent condition (*p* < 0.05). On the other hand, in the audio-visual-incongruent condition, the rate of audio (correct) responses for no image was higher than for the other types of presentation (*p* < 0.05), and the audio response for the full-face presentation was lower than that for either the mouth or mouth and eyes presentation (*p* < 0.05). These results suggested that any type of visual speech cue improved the perception accuracy for audio-visual-congruent stimuli. In addition, as we expected, the influence of a visual speech cue on perceiving a voice was strongest for the presentation of full-face speech with the incongruent voice.

Next, we examined the relationship between the AQ scores and the effect of visual presentation on speech perception. The scores on the AQ ranged from 10 to 43 with a mean of 21.2 (*SD* = 6.07). In order to examine the relationship between task performance and AQ scores, we calculated Pearson correlation coefficients (see Supplementary Material). No significant correlation was observed in the audio-visual-congruent condition. In the audio-visual-incongruent condition, AQ scores were significantly positively correlated with the audio (/pa/) response, *r*_(50)_ = 0.31, *p* < 0.05, and negatively correlated with the fused (/ta/) response, *r*_(50)_ = −0.31, *p* < 0.05, but only for the full-face presentation. These correlations replicated the results in Experiment 1, which indicates that individuals with high AQ scores showed less of a visually captured response than individuals with low AQ scores did, although this only occurred in the full-face incongruent speech condition.

**Figure 4:**
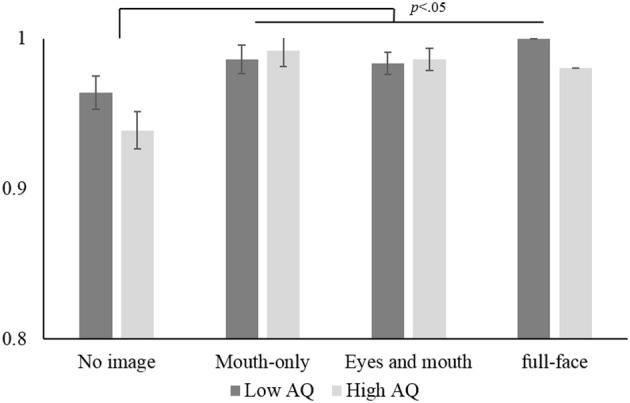
**Mean accuracy for the audio-visual-congruent stimuli in the low-AQ and high–AQ groups**. Mean accuracy is the mean rate of correct responses for the three syllables. Error bars indicate standard errors.

Then, we examined group differences between the high-AQ and low-AQ groups in each condition. From among the participants, we picked 10 with scores of 27 or over as the former (2 males and 8 females, mean AQ score = 30.1), and another 10 with scores of 16 or under as the latter (5 males and 5 females, mean AQ score = 13.7). A between-groups *t*-test showed a significant difference in the AQ scores, *t*_(18)_ = 10.28, *p* < 0.01, *r* = 0.92. In the congruent condition (see Figure 4), a mixed ANOVA revealed that the main effect of visual presentation was significant, *F*_(3, 54)_ = 11.42, *p* < 0.01, partial η^2^ = 0.36, but that the main effect of groups and the one-way interaction were not.

**Figure 5:**
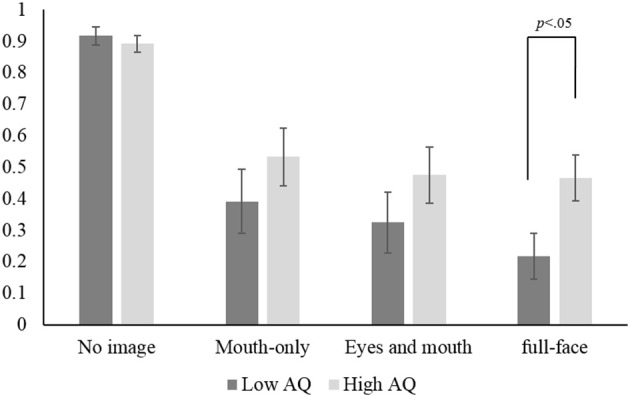
**Mean audio response to the audio-visual-incongruent stimuli in the low-AQ and high–AQ groups**. Error bars indicate standard errors.

For audio (correct) responses in the incongruent condition (see Figure 5), although no significant main effect of groups was found, there was a significant main effect of visual presentation, *F*_(3, 54)_ = 63.83, *p* < 0.01, partial η^2^ = 0.75, and a significant one-way interaction, *F*_(3, 54)_ = 3.16, *p* < 0.05, partial η^2^ = 0.15. This interaction revealed that the simple main effect of groups was significant only in the full-face presentation condition, *F*_(1, 18)_ = 5.37, *p* < 0.01, partial η^2^ = 0.23, so that the individual differences between the high- and low-AQ groups appeared only in the full-face presentation condition. We also found that the effect size of visual presentation was slightly smaller in the high-AQ group, *F*_(3, 54)_ = 19.88, *p* < 0.01, partial η^2^ = 0.72, than in the low-AQ group, *F*_(3, 54)_ = 47.11, *p* < 0.01, partial η^2^ = 0.52. Similar results were found for fused responses in the incongruent condition, i.e., a main effect of visual presentation, *F*_(3, 54)_ = 67.49, *p* < 0.01, partial η^2^ = 0.76, and a significant one-way interaction, *F*_(3, 54)_ = 3.14, *p* < 0.05, partial η^2^ = 0.15. These results indicate that the effect of global facial information was greater in the low-AQ group than in the high-AQ group, although this effect was found in both groups.

### Discussion

In Experiment 2, we aimed to investigate the effect of global facial information on audio-visual speech perception, and its relationship with level of autistic traits. With regard to the former purpose, we hypothesized that audio responses would be observed less frequently for the full-face image of a visual speech cue than for the only-mouth image of a visual speech cue in the incongruent condition. As we expected, our results revealed that the rate of audio responses was lower for the full-face image of a visual speech cue than for the other three types of visual speech cue in the audio-visual-incongruent condition. This indicates that global facial information enhances the influence of a visual cue on perceiving a voice. Unlike previous results (e.g., Rosenblum et al., 2000; Hietanen et al., 2001), the difference in visual influence between the full-face presentation and the only-mouth presentation was robust in our study. Our result directly supports the assumption that the processing of global facial information (extraoral region) might be used for audio-visual speech integration (e.g., Thomas and Jordan, 2004; Eskelund et al., 2015).

With regard to the relationship with autistic traits, our results showed that the individual differences between the high-and low-AQ groups appeared only when a full-face image of a visual speech cue with an incongruent voice was presented. Such a group difference was not found in accuracies for the audio-visual-congruent stimuli. Furthermore, the effect of global facial information in the McGurk effect was small in the high-AQ group. This indicates that the local bias on face processing might play a role in audio-visual speech perception, as well as in the recognition of facial identity (Joseph and Tanaka, 2003; Deruelle et al., 2004) and of facial expression (Kätsyri et al., 2008). These results suggest that the visual influence on perceiving voice was weaker in individuals with high AQ scores than in those with low AQ scores because of the weakness of processing global facial information in the McGurk effect.

## General discussion

### Implications for the dimensional model of autism spectrum disorder

In two experiments, we examined the link between level of autistic traits and individual differences in audio-visual speech perception. The results demonstrated that level of autistic traits did not correlate with the accuracy for perceiving audio-visual-congruent speech, regardless of the visual speech presentation condition. Moreover, we did not find a correlation between level of autistic traits and the accuracy for perceiving auditory speech, although individual differences in the audio-visual-incongruent condition, in which the McGurk effect was observed, were related to the degree of autistic traits in the general population. In the audio-visual-incongruent condition, individuals with high AQ scores showed fewer occurrences of the McGurk effect than individuals with low AQ scores did. These results indicate that autistic traits only correlated with the strength of visual influence on perceiving a voice in the audio-visual-incongruent condition.

Our findings have important implications for the dimensional model of ASD, especially for analog studies investigating symptoms of ASD in the general population. With regard to the influence of visual speech cues on perceiving a voice, our results are consistent with those of several previous studies on ASD (De Gelder et al., 1991; Williams et al., 2004; Saalasti et al., 2011; Stevenson et al., 2014). Such atypical processing in individuals with high AQ scores has been reported in the context of perceptual learning (Reed et al., 2011), perspective-taking (Brunye et al., 2012), and lexical effects on speech perception (Stewart and Ota, 2008). As with these studies, our results also support the dimensional model of ASD.

We considered that previous results were mixed due to the heterogeneity of a clinical ASD population in the profile of hyper- and hypo-sensitivity (Woynaroski et al., 2013). To eliminate this factor, we adopted an analog design and used a sample of individuals with TD, who were free from problems with sensory inputs. As we expected, we found significant relationships between level of autistic traits and individual differences in audio-visual speech perception. Some studies showed that it was possible to control the influence of other factors, such as the Big Five personality traits (Wakabayashi et al., 2006a), schizotypal personality (Wakabayashi et al., 2012), and degree of hyper- or hypo-sensitivity (Ujiie and Wakabayashi, 2015). These indicate that an analog design might be an effective approach in the investigation of ASD symptoms to control factors other than the degree of autistic traits.

### The role of global facial information in the occurrence of the McGurk effect

Our results showed a more robust effect of global facial information in the occurrence of the McGurk effect, as compared to previous studies (e.g., Rosenblum et al., 2000; Hietanen et al., 2001). This indicates that extraoral region of visual speech cues might be used for audio-visual speech integration (Thomas and Jordan, 2004). Our results, however, could not reveal whether global facial information is critical for audio-visual speech perception. One previous study (Jordan and Thomas, 2011) stated that the mouth region of a visual speech cue is important for audio-visual speech perception, which is something we also found in this study, although the extraoral region could also be used. In this study, global facial information did not have a strong effect on audio-visual-congruent speech perception. This means that the accuracy when hearing a voice increased when any type of visual speech cue was presented with a congruent voice, compared to when only a voice was presented. Moreover, in the incongruent condition, the influence of a visual speech sound appeared even when a voice with an incongruent visual speech cue of only a mouth was exhibited. These findings indicate that information provided by the mouth region is more critical for audio-visual speech perception, than is global facial information.

An issue in this study is that we did not consider the unnaturalness of the stimulation presentation. In this study, a mouth image included a range of motion of the upper lip and lower lip. This image of a visual speech cue was either presented on black background or presented along with other facial parts (eyes or full face). Jordan and Thomas (2011) pointed out that a display that does not obscure all of the face except for the mouth was unnatural. Therefore, rather than global face processing, this unnaturalness might have been what influenced audio-visual speech perception. Another issue is that we used only one combination of visual and audio syllables in the incongruent condition. Previous studies have shown that the effect of global face processing varied with the stimulus, such as a different talker or a different combination of syllables (Jordan and Bevan, 1997; Rosenblum et al., 2000). Nevertheless, the number of talkers in our stimuli (six talkers) was relatively larger than that used in previous studies (Jordan and Bevan, 1997; Rosenblum et al., 2000), as was our sample size.

### The relationship between level of autistic traits and local bias in the McGurk effect

With regard to the local bias exhibited by individuals with ASD, our results suggested a link between level of autistic traits and a weak ability to process global facial information in McGurk stimuli. In our results, the effect of global facial information in the McGurk stimuli was found to be smaller in individuals with low AQ scores than in individuals with high AQ scores, who show less likelihood of the McGurk effect occurring. This could be interpreted as indicating that individuals with high AQ scores show a local bias toward a visual speech cue and that their weak ability to process global facial information leads to individual differences in the McGurk effect. On the other hand, it is possible that other factors might have influenced our results, such as the atypical processing of global motion (Koldewyn et al., 2010), of visual attention (Zhao et al., 2013), or of gaze behavior (Klin et al., 2002).

Previous results provide us with some advantage to understand the influence of gaze behavior in our study. It has been shown that individuals with ASD exhibit atypical gaze behavior toward faces when they observe face stimuli (see, for a review, Senju and Johnson, 2009). Klin et al. (2002) indicated that individuals with ASD tend to fixate more on the mouth region when a dynamic face is presented, while individuals without ASD tend to fixate more on the region of the eyes. However, Saalasti et al. (2012) reported that no differences in gaze behavior between adults with ASD and controls that could have accounted for the individual differences in the McGurk effect. Moreover, Paré et al. (2003) showed that gaze fixations within the talker's face, which meant that gaze was fixed on the talker's mouth or on the talker's eyes, did not influence the likelihood of the McGurk effect occurring in adults with TD. Thus, it could be considered that, even if gaze behavior during trials differed between the high-AQ and low-AQ groups in Experiment 2, such differences would not have substantially influenced the results of this study.

As another limitation in this study, it was unclear whether the individual differences in lip-reading were related to individual differences in the McGurk effect, because we did not use visual-only stimuli in this experiment. Some studies have reported that individuals with ASD experience a deficit in perceiving audio-visual speech because of their poor ability to lip-read (Williams et al., 2004; Woynaroski et al., 2013). Therefore, if individuals with high AQ scores have difficulties in lip-reading, individual differences in the McGurk effect might be caused by poor lip-reading ability, rather than by a local bias toward a visual speech cue. Nevertheless, the results of Experiment 2 showed a significant main effect of visual presentation in the congruent condition for both the high-AQ and low-AQ groups. In other words, when any type of congruent visual speech cue was exhibited, improved accuracy for perceiving a voice was found regardless of level of AQ. If the high-AQ group in this study had difficulties in lip-reading, such improvement would not have been found in that group. In order to clarify the role of a local bias toward a visual speech cue during audio-visual speech perception, these factors should be investigated directly in further studies.

## Conclusion

In conclusion, level of the autistic traits in the general population was found to correlate negatively with visually influenced percepts with the McGurk stimuli. This is the first report of such a correlation. Moreover, individuals with high levels of autistic traits showed a weak ability to process global facial information during the McGurk stimuli.

### Conflict of interest statement

The authors declare that the research was conducted in the absence of any commercial or financial relationships that could be construed as a potential conflict of interest.
