# Supplementary material for: The relationship between level of autistic traits and local bias in the context of the McGurk effect
Source: Front Psychol. 2015 Jun 30;6:891. doi: 10.3389/fpsyg.2015.00891 (PMC4484977; doi:10.3389/fpsyg.2015.00891)
Supplement: Supplementary file 1 [file DataSheet1.DOCX]

***Supplementary Figures and Tables***

**The relationship between level of autistic traits and local bias in the context of the McGurk effect.**

**Yuta Ujiie^1,2^*, Tomohisa Asai^3^, Akio Wakabayashi^4^**

^1^ Graduate School of Advanced Integration Science, Chiba University, Chiba, Japan

^2^ Japan Society for the Promotion of Science, Tokyo, Japan

^3^ NTT Communication Science Laboratories, NTT Corporation, Kanagawa, Japan

^4^ Faculty of Letters, Chiba University, Chiba, Japan

*** Correspondence:** Yuta Ujiie, Graduate School of Advanced Integration Science, Chiba University, 1-33 Yayoi-cho, Inage, Chiba 263-8522, Japan.

Email: [chiba_psyc_individual@yahoo.co.jp](mailto:chiba_psyc_individual@yahoo.co.jp)

1. **Supplementary Figures**

Supplementary Figure 1 (File name: “Image 1”): Mean accuracies for the audio-visual congruent stimuli and audio-only stimuli in the groups of low AQ and high AQ (experiment 1).Error bars mean standard errors.

1. **Supplementary Tables**

Supplementary Table 1. Correlations betweenAQ scores and correct responses in the audio-visual congruent condition (experiment 2).

|  | Mean | SD | Correlations with the AQ scores |
| --- | --- | --- | --- |
| No image (audio only) | .96 | .04 | -.16 |
| Mouth-only | .99 | .02 | -.05 |
| Eyes and mouth | .99 | .02 | -.05 |
| Full-face image | .98 | .05 | -.09 |

Note: N = 50. AQ: Autism-spectrum Quotient.

Supplementary Table 2. Correlations between AQ scores and each responses in the audio-visual incongruent condition (experiment 2).

|  | Mean | SD | Correlations with the AQ scores |
| --- | --- | --- | --- |
| No image (audio only) |  |  |  |
| Audio response (/pa/) | .91 | .01 | -.09 |
| Fused response (/ta/) | .07 | .09 | .11 |
| Visual response (/ka/) | .02 | .06 | -.03 |
| Mouth-only |  |  |  |
| Audio response (/pa/) | .51 | .29 | .17 |
| Fused response (/ta/) | .47 | .28 | -.14 |
| Visual response (/ka/) | .03 | .06 | -.13 |
| Eyes and mouth |  |  |  |
| Audio response (/pa/) | .46 | .28 | .15 |
| Fused response (/ta/) | .51 | .27 | -.12 |
| Visual response (/ka/) | .03 | .08 | -.12 |
| Full-face image |  |  |  |
| Audio response (/pa/) | .35 | .26 | .31 * |
| Fused response (/ta/) | .60 | .26 | -.31 * |
| Visual response (/ka/) | .05 | .12 | .00 |

Note: N = 50. AQ: Autism-spectrum Quotient.
