# Supplementary figures and images for: The relationship between level of autistic traits and local bias in the context of the McGurk effect
Source: Front Psychol. 2015 Jun 30;6:891. doi: 10.3389/fpsyg.2015.00891 (PMC4484977; doi:10.3389/fpsyg.2015.00891)

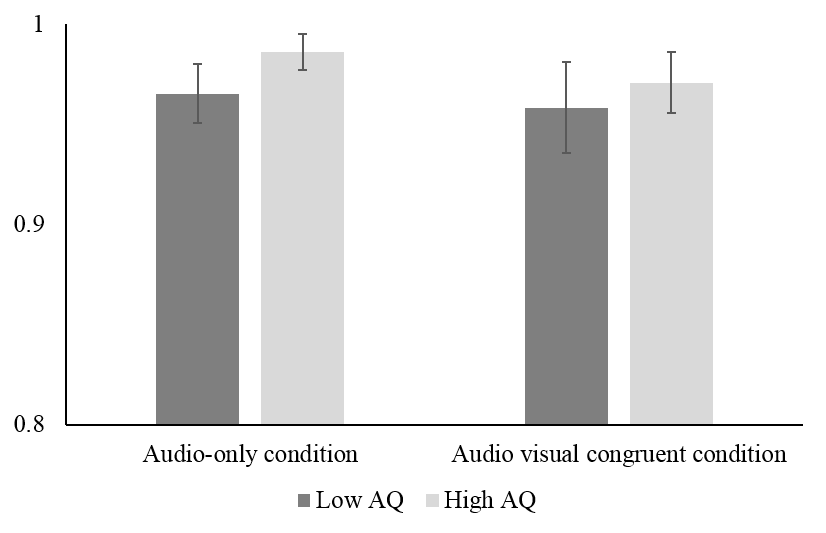

Supplement: Supplementary file 2 [file Image1.TIF]
